# Candiduria in hospitalized patients in teaching hospitals of Ahvaz

**Published:** 2012-12

**Authors:** A Zarei-Mahmoudabadi, M Zarrin, F Ghanatir, B Vazirianzadeh

**Affiliations:** 1 Department of Medical Mycology, School of Medicine, Ahvaz Jundishapur University of Medical Sciences, Ahvaz, Iran; 2 Infectious Diseases and Tropical Medicine Centre, Ahvaz Jundishapur University of Medical Sciences, Ahvaz, Iran; 3 Department of Medical Entomology, School of Public Health, Ahvaz Jundishapur University of Medical Sciences, Ahvaz, Iran

**Keywords:** Candiduria, Renal infection, *Candida albicans*, Nosocomial infections, Risk factors

## Abstract

**Background and Objectives:**

Nosocomial infections are usually acquired during hospitalization. Fungal infection of the urinary tract is increasing due to predisposing factors such as; antibacterial agents, indwelling urinary catheters, diabetes mellitus, long hospitalization, immunosuppressive agents, use of IV catheters, radiation therapy, malignancy. The aim of our study was to determine the prevalence of candiduria and urinary tract infection in patients admitted in Golestan and Emam Khomeini hospitals of Ahvaz, Iran.

**Materials and Methods:**

During 14 months, a total of 744 urine samples were collected and transferred to medical mycology laboratory immediately. Ten µl of uncentrifuged sample was cultured on CHROM agar Candida plates and incubated at 37°C for 24-48h aerobically. *Candida* species were identified based on colony morphology on CHROM agar Candida, germ tube production and micro-morphology on corn meal agar including 1% Tween 80.

**Results:**

In the present study, 744 hospitalized patients were sampled (49.5%, female; 50.5%, male). The prevalence of candiduria in subjects was 16.5% that included 65.1% female and 34.9% male. The most common isolates were *C. albicans* (53.3%), followed by *C. glabrata* (24.4%), *C. tropicalis* (3.7%), *C. krusei* (2.2%), and *Geotrichum* spp. (0.7%) Urine cultures yielded more than 10,000 yeast colonies in 34.1% of cases, and the major predisposing factor associated with candiduria was antibiotic therapy (69.1%).

**Conclusion:**

Candiduria is relatively common in hospitalized patients in educational hospitals of Ahvaz. In addition, there is a strong correlation between the incidence of candiduria in hospitalized patients and broad-spectrum antibiotics therapy.

## INTRODUCTION

The urinary tract is the main site of nosocomial infection among hospitalized patients. Nearly 90% of urinary tract infections (UTIs) are caused by bacteria and only 10-15% are caused by fungi (funguria) (1, 2). *Candida* species are the most commonly identified fungi in urine and candiduria is the presence of *Candida* species in the urine (3). Candiduria may indicate bladder colonization due to indwelling catheters, fungal bezoar, and primary or disseminated candidiasis (3, 4, 5). Asymptomatic candiduria is usually a benign and transient condition in many patients and does not require systemic antifungal therapy (6). However, disease in immunocompromised patients has a high risk of morbidity and mortality (7). There are no clear criteria for differentiating between colonization and infection or between upper and lower UTI.

The disease has increased significantly in the past decades due to new procedures for patients, such as chemotherapy, urinary indwelling catheters, nephrostomy tubes, immunosuppressive therapy, hemodialysis, previous surgery, long term and broad spectrum antimicrobial usage and renal transplantation (6, 8, 9). Reports show that due to use of broad-spectrum antibiotics, candiduria is increasing in extended hospitalized patients in many teaching hospitals (8–10). In addition, elderly, gender, diabetes mellitus, urinary tract abnormality, chronic renal failure, malignancy and neutropenia were also reported as candiduria risk factors (8, 9, 11).

According to the United State of America National Nosocomial Infection Surveillance systems, *Candida* species are the 7^th^ most common nosocomial pathogens (12). Although the vast majority of candiduria are caused by *Candida albicans*, non-*albicans* such as *C. glabrata* and *C. tropicalis* are emerging as nosocomial pathogens for UTIs (5, 6, 10, 13). The aim of the present study was to investigate the incidence of risk factors associated with candiduria in hospitalized patients in two educational hospitals in Ahvaz.

## MATERIALS AND METHODS

During the period 2010-2011, a total of 744 hospitalized patients in two educational hospitals, Golestan and Emam Khomeini in Ahvaz, Iran, were considered. Data collected for each patient included; gender, age, duration of hospitalization, catheter use, antimicrobial therapy, diabetes mellitus and hospital ward. The aim of research was clearly explained to patients. All patients agreed to participate in this study and signed inform consent which was approved by the Ahvaz Jundishapur University of Medical Sciences ethics committee. None of the patients used antifungal drugs during the sampling. However, they used some antibacterial agents such as chloramphenicol, tetracycline and amikacin.

Urine samples were collected from patients into sterile urine bottles in the morning and immediately transferred to medical mycology laboratory for diagnostic tests. Ten µl each of uncentrifuged urine sample was cultured on CHROM agar Candida (CHROM agar Candida^®^, France) plates as lawn and incubated at 37°C for 24-48h aerobically. The number of colonies on each plate were counted and recorded based on colony colors. Then, a direct smear was prepared from each colony and confirmed as yeasts. Yeasts were identified based on colony morphology on CHROM agar Candida, germ tube production and micro-morphology on corn meal agar including 1% tween 80. All *C. albicans* isolates were transferred on Sabourauds dextrose agar (SDA) and kept at 45°C for 48 h. In contrast to *C. albicans*, *C. dubliniensis* does not have the ability to develop at 45°C.

## RESULTS

In the present study, 744 hospitalized patients in two education hospitals in Ahvaz, capital city of Khuzestan province, were sampled (368, 49.5%, female; 376, 50.5%, male). Table 1 shows distribution of patients in different departments in both hospitals. As shown the most hospitalized patients were in internal medicine, gynecology and obstetrics and surgery departments respectively. The highest frequency of candiduria in VIP was 50%, followed by ICU/CCU 39/1%, psychology 30.0% and gynecology and obstetric 25.2%. Whereas, the lower frequency of candiduria was found in the department of hematology (4.8%) (Table 2). Table 3 shows the details positive hospitalized patients in different departments in both hospitals. Out of 123 positive cases, 54.5% related to two departments of internal medicine (30.9%) and genecology and obstetrics (23.6%). In our study, the risk factors in candidura patients were: antibiotic therapy (69.1%), using fully cathater (18.7%) and type 2 diabetes (12.2%).

**Table 1:** The distribution of 744 hospitalized patients in different departments in two educational hospitals.

Departments	Male		Female		Total	

	No	%	No	%	No	%
Internal medicine	113	15.2	98	13.2	211	28.4
Genecology and obstetric	0	0.0	115	15.5	115	15.5
Surgery	68	9.1	24	3.2	92	12.4
Urology	50	6.7	8	1.1	58	7.8
Neurology	26	3.5	26	3.5	54	7.3
ENT	32	4.3	21	2.9	53	7.1
Orthopedic	37	5.0	13	1.7	50	6.7
ICU, CCU	9	1.2	14	1.9	23	3.1
Hematology	13	1.7	8	1.1	21	2.8
Dermatology	7	0.9	10	1.3	17	2.3
Ophthalmology	8	1.1	4	0.5	12	1.6
Psychology	0	0.0	10	1.3	10	1.3
Cardiology	4	0.5	6	0.8	10	1.3
Infants	6	0.8	4	0.5	10	1.3
Physical medicine	1	0.1	3	0.4	4	0.5
VIP	0	0.0	4	0.5	4	0.5
Total	376	50.5	368	49.5	744	100

**Table 2:** The frequency of candiduria in different departments.

Departments	Sampled patients	Positive patients	

		No	%
VIP	4	2	50.0
ICU, CCU	23	9	39.1
Psychology	10	3	30.0
Genecology and obstetric	115	29	25.2
Internal medicine	211	38	18.0
Dermatology	17	3	17.6
Urology	58	10	17.2
Orthopedic	50	6	12.0
ENT	53	6	11.3
Neurology	54	6	11.1
Infants	10	1	10.0
Surgery	92	8	8.7
Ophthalmology	12	1	8.3
Hematology	21	1	4.8

**Table 3:** The distribution of 123 positive cases of candiduria in different departments in two educational hospitals.

Department	Male		Female		Total	

	No	%	No	%	No	%
Internal medicine	18	14.6	20	16.3	38	30.9
Genecology and obstetric	0	0.0	29	23.6	29	23.6
Urology	6	4.9	4	3.3	10	8.1
ICU, CCU	7	5.7	2	1.6	9	7.3
Surgery	5	4.1	3	2.4	8	6.5
Neurology	2	1.6	4	3.3	6	4.9
Orthopedic	4	3.3	2	1.6	6	4.9
ENT	1	0.8	5	4.1	6	4.9
Dermatology	0	0.0	3	2.4	3	2.4
Psychology	0	0.0	3	2.4	3	2.4
VIP	0	0.0	2	1.6	2	1.6
Hematology	0	0.0	1	0.8	1	0.8
Ophthalmology	0	0.0	1	0.8	1	0.8
Infants	0	0.0	1	0.8	1	0.8
**Total**	43	35	80	65	123	100

In the present study, the age range of hospitalized patients was eight months to 90 years. As shown in Table 4, the most hospitalized patients had 21-25 year olds, whereas only 2.0% of patients ranged between 11-15 year olds. The prevalence of candiduria in considered hospitalized patients was 16.5% (123 of 744) that 65% were female and 35% male. The most positive case was included in age range 36-65 years (44.7%) followed by; 41.5% > 35 year, and 13.8% < 66 year. In this study the length of hospitalization was 4-10 days for 48.0% (59/123) of the patients, followed by 39.8% (49/123), < 3 days and 12.2% (15/123) >10 days. *C. albicans* was the most common species (53.3%) fallowed by *C. glabrata* (24.4%), *C. tropicalis* (3.7%), *C. krusei* (2.2%), *Geotrichum* spp. (0.7) and 15.6% were unspecified (*Candida* spp.). Eight and two patients, two and three *Candida* species were respectively isolated concurrently. Urine cultures yielded heavy candidal growth, more than 10^4^CFU/ml in 34.1% of cases, followed by 5001-10000 (5.9%), 1001-5000 (19.3%) and lower than 1000 (40.7%). Table 5 shows the details of colony counts for each isolated *Candida*.

**Table 4:** The distribution of the age range of 744 hospitalized patients in two educational hospitals.

Age range (year)	Male		Female		Total	

	No	%	No	%	No	%
>10	14	1.9	6	0.8	20	2.7
11-15	4	0.5	11	1.5	15	2.0
16-20	44	5.9	25	3.4	69	9.3
21-25	55	7.4	41	5.5	96	12.9
26-30	24	3.2	42	5.6	66	8.9
31-35	18	2.4	44	5.9	62	8.3
36-40	23	3.1	31	4.2	54	7.3
41-45	18	2.4	18	2.4	36	4.8
46-50	40	5.4	24	3.2	64	8.6
51-55	22	3.0	20	2.7	42	5.6
56-60	25	3.4	20	2.7	45	6.0
61-65	17	2.3	20	2.7	37	5.0
66-70	16	2.2	15	2.0	31	4.2
71-75	16	2.2	11	1.5	27	3.6
76-80	16	2.2	15	2.0	31	4.2
<80	23	3.1	15	2.0	38	5.1
**Total**	376	50.5	368	49.5	744	100

**Table 5:** Colony count of each isolated species.

Colony count	>1000 (CFU)	1001-5000	5001-10000	<10000	Total
*C. albicans*	33	12	5	22	72(53.3%)
*C. glabrata*	13	6	1	13	33(24.4%)
*C. tropicalis*	0	1	1	3	5(3.7%)
*C. krusei*	0	1	0	2	3(2.2%)
*Geotrichum* spp.	0	0	0	1	1(0.7%)
*Candida* spp.	9	6	1	5	21(15.6%)
Total	55(40.7%)	26(19.3%)	8(5.9%)	46(34.1%)	135(100%)

In the present study, *Candida* growth in urine was broadly associated with antibiotic therapy 85(69.1%), followed by Foley catheters 21(17.1%), diabetes mellitus 14(11.4%) and 3(2.4%) of patients were without any risk factors.

## DISCUSSION

Candiduria accounted for up to 10% of UTIs and has resulted in increased rate of mortality during the last decade due to use of new medical instruments, new treatments, surgery and transplantation (1, 2, 14). In addition, Weinberger *et al*. (10) believe that hospitalization in teaching hospitals increase candiduria in patients which correlate with the amount of departmental antibiotic consumption. Candiduria is rare in healthy people but relatively frequent in hospitalized patients (2, 10). In our study, 16.5% of culture from sampled patients yielded different species of *Candida*. Our hospitals (Golestan and Emam Khomeini) are two crowded reference hospitals in Khuzestan province. Other studies have documented that hospitalized patients are relatively susceptible to candiduria (2, 5, 15). In a study in Brazil, 22% of urine samples from hospitalized patients were positive for *Candida* species (5). Several reports show that the frequency of candiduria in women is more than men (5, 9). We observed that the male:female ratio in our series is 80:43, contrary to the male predominance reported in the study by Paul *et al*. (14). In addition, extended hospitalization in 48.0% of our patients with candiduria was between 4 to 10 days. In our study candiduria were also more prevalent in age range 36-65 years (44.7%) followed by; 41.5% > 35 year, and 13.8% < 66 year.

Nayman Alpat *et al*. (1) had believed that long duration of ICU and hospital stay increase the prevalence of candiduria in patients. In addition, candiduria in ICU patients is a marker for increased mortality (16). In a study conducted by Sellami *et al*., (11) 56 of hospitalized patients in the ICU department (34%) were positive for candiduria. In our study 50% of hospitalized patients in special (VIP) ward had positive culture for *Candida* followed by, ICU/CCU 39/1%, psychology 30.0% and gynecology and obstetrics 25.2% (Table 4). Antibiotics therapy, urinary catheterization and surgical procedure are the most common risk factors for candiduria in hospitalized patients (3, 5, 6, 10). 69.1% of our patients with candiduria consumed different antibiotics. Whereas use of the Foley catheter and diabetes mellitus are only associated with candiduria in 28.5%.

*C. albicans* had remained the major agents of candiduria until recently (1, 2, 10), however several reports show that non-albicans species, especially *C. tropicalis* and *C. glabrata* now predominate in many regions (2, 17). Non-*albicans* species accounted for 71% and 64.4% of isolates in Paul *et al*. (14) and Kobayashi *et al*. (5) reports, respectively. Although the majority of candiduria in the present study are caused by *C. albicans* (53.3%), non-*albicans* species, especially *C. glabrata* (24.4%) is emerging as a nosocomial infection. In our study, eight patients demonstrated two *Candida* species and two patients demonstrated three *Candida* species. Previously, Weinberger *et al*. (10) reported isolates in hospitalized patients. Several reports from Iran showed the Candida colonization in the urinary tract (18–20). Mardani *et al*. reported a case of Candida cystitis in a patient with diabetes mellitus (21). UTIs due to *C. glabrata* have recently increased and these infections are usually resistant to fluconazole (7, 6). There are several criteria to diagnose candiduria, for example in adults, CFU criteria range from 10^3^-10^5^ CFU/ml of urine, whereas National Institutes of Health defined lower CFU cutoff (10^3^) (9). In our study, 59.3% of patients with positive culture had CFUs of more than 10^3^.

In conclusion, candiduria is relatively common in hospitalized patients in educational hospitals of Ahvaz and more than half (50.5%) of positive cases were hospitalized in the internal medicine department. However, the frequency of positive candiduria in VIP patients was 50% (2 of 4), ICU/CCU 39.1% (9 of 23), psychology 30.0% (3 of 10) and gynecology and obstetrics 25.2% (29 of 115). In addition, there is the strong correlation between the incidence of candiduria in hospitalized patients and broad-spectrum antibiotics therapy. In addition, these results are useful for control of candiduria in hospitalized patients.
